# Acoustic-Based Screening Method for the Detection of Total Aflatoxin in Corn and Biological Detoxification in Bioethanol Production

**DOI:** 10.3389/fmicb.2020.00543

**Published:** 2020-04-15

**Authors:** Grazina Juodeikiene, Darius Cernauskas, Karolina Trakselyte-Rupsiene, Elena Bartkiene, Daiva Zadeike, Greta Banyte, Antonello Santini

**Affiliations:** ^1^Department of Food Science and Technology, Kaunas University of Technology, Kaunas, Lithuania; ^2^Food Institute, Kaunas University of Technology, Kaunas, Lithuania; ^3^Institute of Animal Rearing Technologies, Lithuanian University of Health Sciences, Kaunas, Lithuania; ^4^Department of Food Safety and Quality, Lithuanian University of Health Sciences, Kaunas, Lithuania; ^5^Department of Pharmacy, University of Napoli Federico II, Naples, Italy

**Keywords:** *Aspergillus* spp., corn, nuts, rapid method, aflatoxins screening, acoustic sensors, bioethanol, detoxification

## Abstract

*Aspergillus* spp. are widely occurring fungi in nature; they produce toxic compounds such as aflatoxins (AFs) and mainly target plant products such as corn and nuts. The development of prevention strategies is challenging because AFs are highly toxic and have been regulated to small concentrations. This study proposes a new strategy of AF prevention through the application of rapid methods using acoustic techniques in combination with fermentation for the elimination of contaminated corn from bioethanol production processes. An acoustic device was used for the analysis of model systems consisting of corn and nuts (hazelnuts and peanuts) contaminated with different amounts of AFs. High correlations were obtained between penetrated acoustic signal amplitude (Ap) and corn sample density, and between Ap and AF content. Also, relationships were found between changes in Ap values and AF contamination in the nuts model systems. The results of biotreatment of contaminated corn during bioethanol production confirmed that AFs cannot be completely eliminated in dried distiller’s grains with solubles, a valuable by-product for animal feed. Microbially, contamination of the raw material has a negative impact on bioethanol quality by increasing the content of volatile compounds. Therefore, the application of methods such as acoustic screening is a promising alternative for rapid AF detection in corn and nuts (it can handle multi-layers of grain). With the application of acoustic techniques, the prevention of AFs in contaminated raw plant materials could be achieved.

## Introduction

*Aspergillus flavus* and *A. parasiticus* are one of the most common fungal strains in the agricultural sector, producing the aflatoxins (AFS) AFB1 and AFB2, and AFG1 and AFG2, which are chemically related to bisfuranocoumarin (Udomkun et al., 2017; He et al., 2018) and found worldwide in soil, air, and plants (Bandyopadhyay et al., 2016; Rushing and Selim, 2019). Aflatoxins are potential carcinogens that frequently contaminate food raw materials such as corn, cottonseed, peanuts, and some tree nuts in high concentrations, representing a high risk to food and feed chains and lowering the nutritional quality (Santini et al., 2009; Mikusova et al., 2010; Ritieni et al., 2010; Weaver et al., 2015; Moretti et al., 2019). Therefore, for the food and feed industry to avoid economical losses, a major task is to control the mycotoxin contamination levels in the end products. The prevention of fungal contamination and the development of methods for the decontamination of foods from mycotoxins are important strategies to protect human and animal health (Ehrlich, 2014; Reinholds et al., 2016; Ismail et al., 2018; Pankaj et al., 2018; Mwakinyali et al., 2019).

The common practice to determine mycotoxins is to use laboratory- and time-intensive fundamental chemical, physical, and enzyme immunoassay analyses (Santos Pereira et al., 2019). Considering the fact that these mycotoxin detection methods are complex and expensive, special attention should be given to innovative mycotoxin determination technology, that will allow quick and cheap detection of mycotoxins in the raw materials. Fungal infection not only results in the accumulation of mycotoxins, but also causes grains to shrivel and become more porous. This phenomenon is known as head blight or scab, one of the indicators of poor wheat grain quality (Juodeikiene et al., 2011; Ropelewska et al., 2019; Zhang and Ji, 2019). Due to changes in grain microstructure a rapid and non-destructive method to evaluate the quality and safety of grains is therefore required to detect and subsequently eliminate these toxins from the food chain. The first portable acoustic device equipped with a wide-range capacity ultrasonic transducer was developed at Kaunas University of Technology (Lithuania) during the implementation of the EUREKA ITEA2 project ACOUSTICS for the prediction of deoxynivalenol (DON) contamination levels in wheat grains. Project results showed that the acoustics method, applied for the first time to grain safety monitoring, is innovative and important in ensuring the safety of grains (ITEA 2 Magazine, 2013; Juodeikiene et al., 2014b). However, until now, no studies of the influence of *Aspergillus* spp. and their metabolites on corn grain and nut microstructure, their technological properties and the use of an acoustic method for the detection of AFS, have been performed.

To avoid the detrimental effects of feed and food contaminated by AFs, not only prevention of contamination but also decontamination of toxic compounds during processing should be applied (Taheur et al., 2019). Fungal infection not only results in the accumulation of AFs, but due to contamination corn raw materials are no longer available for food or feed consumption and can be used as biomass. One of the possible applications of corn biomass is for the production of bioethanol. It is known that fermentation processes could eliminate grain contamination, with the possibility of using the by-products obtained as feed for cattle (Čolović et al., 2019). For this reason, the degradation or decontamination of mycotoxins using appropriate biological microorganisms have been used in the last decade (Juodeikiene et al., 2012; Oliveira et al., 2013; Peles et al., 2019). Recently, the novel aspects of the biological detoxification of mycotoxins (Vila-Donat et al., 2018) included a strategy that relies on mycotoxin inactivation or transformation to non-toxic products by applying low-cost and economically feasible decontamination technologies, retaining the nutritive quality of feed or food, remaining palatable, and not changing significantly the physical properties of the raw material (Haque et al., 2020). A number of microbial species of bacterial and fungal origin have shown the capability to degrade mycotoxins via sorption/enzymatic degradation (Risa et al., 2017; Wang et al., 2018, 2019).

In the present study, the efficiency of corn biomass bioprocessing was explored by using a multi-step prevention system: in the first step, acoustic screening of grains with the elimination of contaminated corn from the production chain was used, and in the second one, a detoxification approach (e.g., fermentation with selected bio-tools) for bioethanol production was applied.

## Materials and Methods

### Plant Material

#### Corn Samples

Uncontaminated corn grains and grains artificially infected with *Aspergillus flavus* with a high level of total AFs (59.2 μg/kg) were obtained from the USDA (United States). The corn model systems were prepared by mixing the uncontaminated corn kernels with 0, 10, 20, 30, 40, 50, 60, 70, 80, 90, and 100% of contaminated kernels.

#### Nut Samples

Additionally, this study analyzed different nuts (peanuts and hazelnuts) obtained from a Lithuanian supermarket. The two model systems (peanuts and hazelnuts) were prepared by mixing whole-appearance nuts (peanuts and hazelnuts) with mold-damaged nuts (0, 10, 20, 30, 40, 50, 60, 70, 80, 90, and 100%). The infected nuts were smaller and more shriveled and were selected according to their degree of shriveling.

### Methods of Analysis

#### Determination of Chemical Composition and the Qualitative Characteristics of Grain or Nuts

Humidity was determined by the weight loss on drying of the grain or nuts (130 ± 3°C) to constant weight [AACC method 44-15 (2000)].

*The bulk density* of the corn or nuts (ρ, kg/m^3^) was calculated using the equation ρ = G/V, where G is mass and V is volume.

#### Grain Microstructure Evaluation

Cross-sectional images of infected and healthy grains were taken by scanning electron microscope EVO 50 (LEO Electron Microscopy Ltd., Cambridge, United Kingdom) equipped with a second electron (SE) detector.

#### Microbiological Contamination of Nuts

Microbiological tests on both types of nuts were carried out using five mixtures: (1) 100% whole nuts, (2) 75% whole nuts + 25% contaminated nuts, (3) 50% whole nuts + 50% contaminated nuts, (4) 25% whole nuts + 75% contaminated nuts, and (5) 100% infected nuts. The total numbers of aerobic microorganisms in the mixtures were determined using the plate-count agar (CM0325, Oxoid, United Kingdom). Nuts (10 g) were mixed with 100 ml of distilled water, and after serial dilution, the obtained homogenate was mixed with the agar medium and incubated at 24°C for 5 days in aerobic conditions. The count of microorganisms was expressed in CFU (colony-forming units) per gram of nuts.

### Acoustic Technique

**FIGURE 1:**
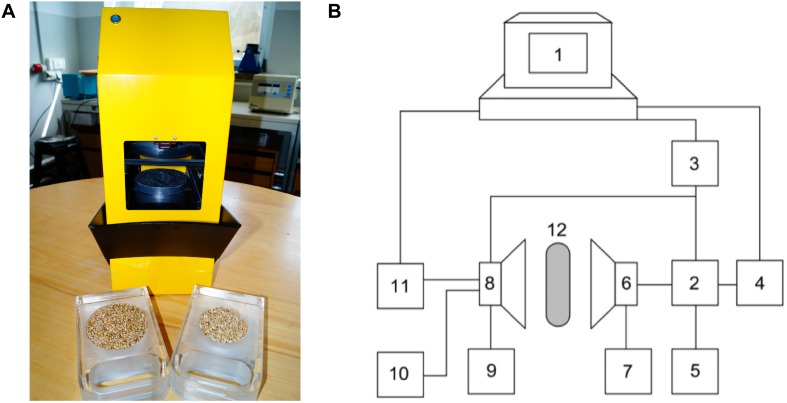
The acoustic spectrometer **(A)** and its schematic drawing **(B)** (1 – computer; 2 – sine-wave generator; 3 – pulse generator; 4 – frequency converter; 5 – frequency meter; 6 – transmitting acoustic aerial; 7 – power supply; 8 – receiving acoustic aerial; 9 – power supply; 10 – oscilloscope; 11 – digital voltmeter; 12 – grain seeds sample under test).

Samples of the corn grain, corn grain model system, and different nuts model systems were screened using a recently developed portable acoustic spectrometer (Figure 1) with penetration (Juodeikiene et al., 2014b).

The spectrometer measures in relative units the amplitude of the acoustic signal (Ap) that penetrates the sample matrix over the frequency range 10–80 kHz. The 15–40 kHz interval was selected as the optimum frequency range. The duration of each measurement was ∼10 s. The test was carried out by placing the test portion of 200 g of sample into a plastic vessel whose base was covered with sound-transmitting material. The thickness of the sample layer was 50 mm and diameter 80 mm.

#### Determination of AFs

The quantitative analysis of the total AFs (B1, B2, G1, and G2) in corn samples and corn samples after bioethanol production (the stillage obtained after drying at 50°C for 24 h) was performed by a competitive enzyme linked immunosorbent assay (ELISA) according to the total AF test (AgraQuant^®^, Romer Labs Ltd., Germany) procedure. The ground test sample amount used in the ELISA assay was 100 g. Mycotoxin extraction and testing was carried out according to the manufacturer’s instructions.

*Acidity analysis* of fermented broth in bioethanol production was performed according to our previous study (Juodeikiene et al., 2012). The concentration of ethanol was determined using direct distillation and pycnometry.

*Volatile compound* determination was completed by gas chromatography (GC). Corn samples with different contamination levels were used for bioethanol production (Table 1). The bioethanol production was performed by using the low-temperature process according to Juodeikiene et al. (2014a). A Hewlett-Packard 5890 gas chromatograph equipped with an FID detector was used for the quantitative analysis of volatile compounds as described by Juodeikiene et al. (2014a).

**TABLE 1:** The corn samples with different contamination levels, used for bioethanol production.

Corn No.	Aflatoxin Concentrations, μg/kg
C-0	0,00
C-14	14,17
C-15	15,24
C-39	38,55
C-50	50,00
C-54	53,68
C-57	56,70

## Results and Discussion

### The Changes in the Microstructural Composition and Microbiological Contamination of Corn Grains and Nuts Damaged by *Aspergillus* spp.

**FIGURE 2:**
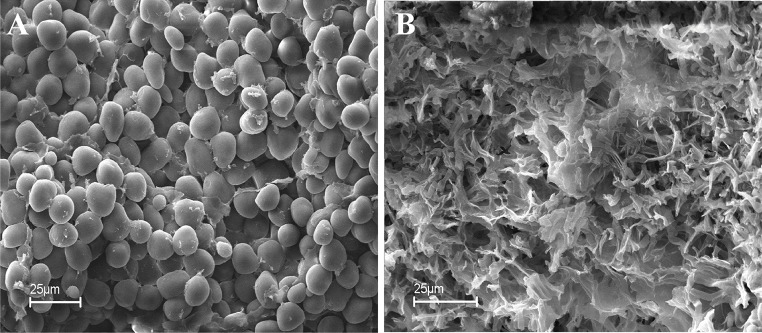
Microscopic view of healthy kernels with intact starch granules **(A)** and kernels damaged by fungi showing stripped starch granules **(B)**.

Microscopic analysis of the grains contaminated by *Aspergillus* and the wholesome corn shows visible damage on the surface of the contaminated grain kernels (Figure 2B) and shows what happens to the structure of the grain kernels when attacked by *Aspergillus* spp. In Figure 2A, the structure of the grain kernel walls is healthy and wholesome; in Figure 2B, the starch granules have been “consumed” by the fungus and a more skeleton-like type of landscape appears. Endosperm cells of healthy kernels (Figure 2A) were filled with regular-shaped starch granules and distributed in the unfolded protein matrix. On the other hand, the endosperm in the *Aspergillus*-damaged kernels were restructured, fractured, and of irregular form and formed single agglomerates that are influenced by the amylolytic degradation of the starch granules (Figure 2B).

**FIGURE 3:**
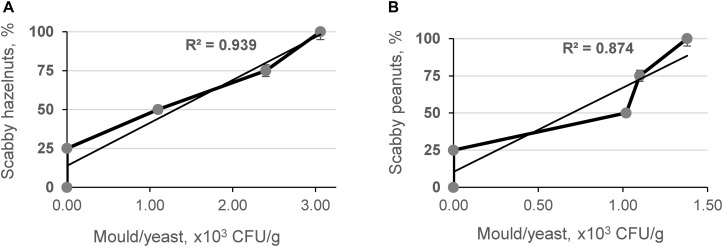
Relationship between content of mold/yeast in scabby hazelnut **(A)** and peanut **(B)** model systems.

Additionally, the microbiological contamination effect was studied on the hazelnut and peanut model systems, which were prepared by mixing visually damaged and contaminated nuts with wholesome ones. Statistical analysis showed a significant positive relationship between the number of scabby hazelnuts (*R*^2^ = 0.939, *p* < 0.05) and peanuts (*R*^2^ = 0.874, *p* < 0.05) in model samples and mold/yeast counts, respectively (Figures 3A,B).

Fungal infection not only results in the accumulation of mycotoxins, but could also considerably influence the structure and the physical criteria of corn grains and nuts. These changes in the structure influence the porosity of the kernels as well as change the packing factor of kernels in the matrix and could be the basis for the development of a screening method for detection of microbial contamination in this type of raw material.

### The Application of the Acoustic Screening Technique for the Detection of Microbiological Contamination in Corn Grains and Nuts Damaged by *Aspergillus* spp.

**FIGURE 4:**
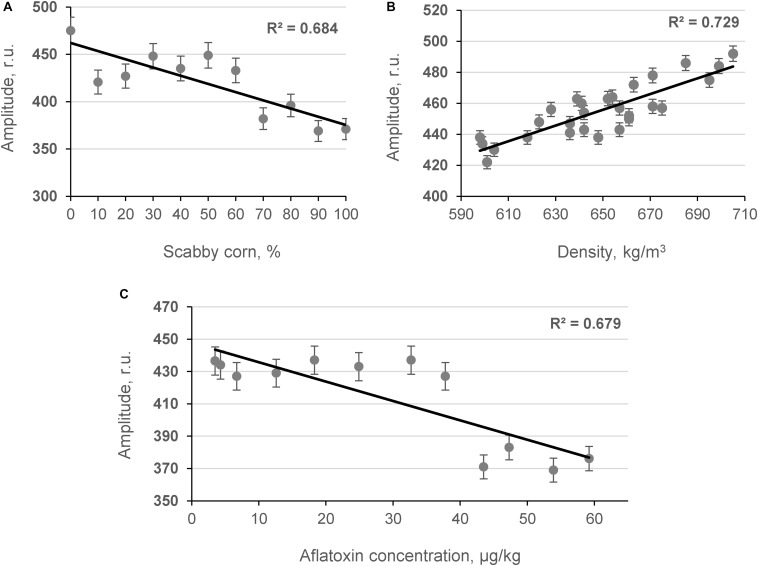
Relationship between content of scabby corn in model systems **(A)**, density of samples **(B),** AF content in samples **(C)**, and amplitude of the penetrated acoustic signal (Ap, frequency 24.3 kHz; *n* = 10).

**FIGURE 5:**
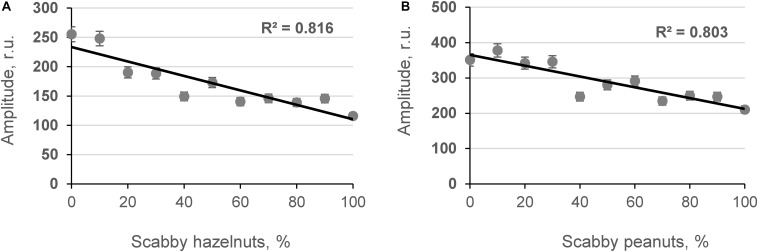
Relationship between content of scabby hazelnuts and peanuts in model systems (**A,B**, respectively) and amplitude of the penetrated acoustic signal (Ap, frequency 18.1 and 27.0 kHz; *n* = 10).

The influence of contaminated grains on grain bulk density was studied by determining the relationship between the content of damaged grains and the amplitude of the penetrating (Ap) acoustic signal measured by the acoustic spectrometer (Figure 4). As shown in Figures 4A,C, strong inverse linear relationships were obtained between the number of contaminated corn grains in model samples, the AF content in the samples measured by ELISA [AFL(ELISA)], and the amplitude of the acoustic signal in the model samples (*R*^2^ = −0.684 and *R*^2^ = −0.679, *p* < 0.05, respectively). A strong positive correlation was observed between the density of model corn samples and the Ap values recorded using the acoustic spectrometer (*R*^2^ = 0.729, *p* < 0.05) as shown in Figure 4B. Further acoustic analyses were performed with contaminated hazelnut and peanut samples (Figures 5A,B).

Results indicated that as microbiological contamination of nuts increased, the amplitude of the acoustic signal penetrating the nut sample decreased. The obtained results are in agreement with previous studies in which a strong dependence between DON and the content of scabby kernels in wheat matrix was found by Juodeikiene et al. (2008, 2014b) and Tutelyan (2004). The results of the fungal invasion are that the attacked grains shrivel (become scabby in the case of wheat) and become more porous. The same is true for corn kernels affected by *A. flavus*, although the shriveling is less manifested compared to wheat grains because the pericarp of the corn kernel is sturdier. At the point of harvest, a mixture of wholesome and shriveled grains (or more porous kernels) is seen. The differences between the wholesome (less scabby nuts) and contaminated nuts (more scabby nuts) can also be detected by screening acoustic techniques (Figure 5). Experiments have shown that the acoustic behavior of porous granular materials can be characterized essentially in terms of porosity and airflow resistance (Guo et al., 2019; Pereira et al., 2019). Furthermore, it was found (Guo et al., 2005) that in beads of cereal grains, the absorption of the acoustic signal depends on the size and shape of the particles. Therefore, it is advisable to use developed equipment at point of harvest where one strain of cereal usually dominates (with one particle size and shape).

### The Effect of Microbial Contamination by *Aspergillus* spp. on the Fermentation Processes During Bioethanol Production

The influence of corn biomass being contaminated with AFs at different levels on alcoholic fermentation was evaluated by chromatographic analysis of the yield ethanol and fusel oils.

**FIGURE 6:**
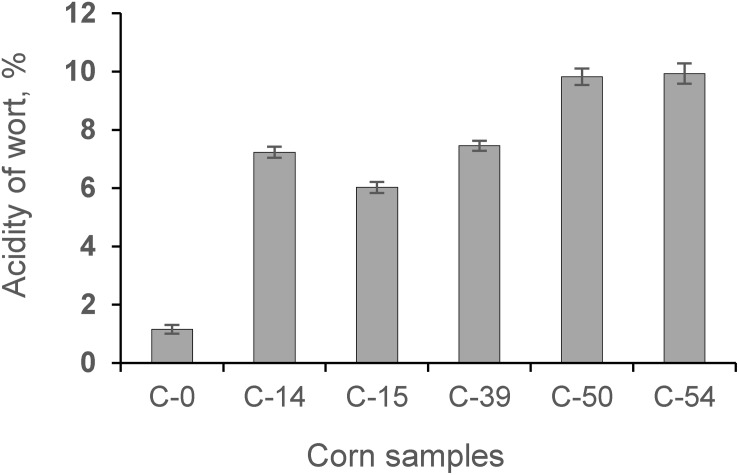
The acidity of wort obtained during fermentation of whole corn grains and contaminated grain samples (C-0, C-14, C-15, C-39, C-50, C-54).

The process of fermentation of corn contaminated with AFs resulted in a higher organic acid formation (on average 7.03 times) in wort compared to the control sample (Figure 6).

**FIGURE 7:**
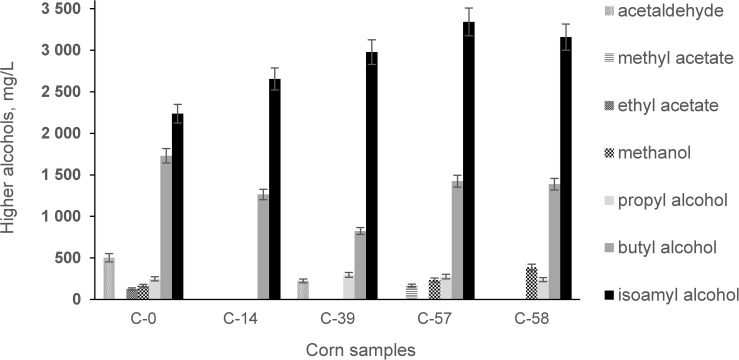
Concentrations of metabolic products in bioethanol during fermentation of corn contaminated with AFs.

**FIGURE 8:**
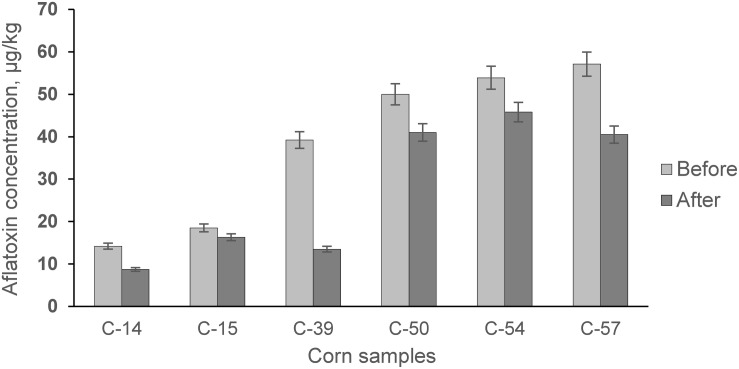
Concentration of AF in corn samples before fermentation versus after fermentation.

Qualitative and quantitative analysis of bioethanol showed (Figure 7) that fermentation of AF-contaminated corn produced, on average, higher levels of higher alcohols: isoamyl alcohol (35.57%), propyl alcohol (8.63%), and methanol (88.62%). During the experiment, the levels of AF in the infected corn before and after fermentation were examined (Figure 8).

Our study showed that contamination of corn with AFs has a negative influence on the fermentation process by increasing the acidity profile (Figure 6) and increasing secondary metabolites in the final product (Figure 7).

However, the positive detoxification effect of fermentation was achieved by reducing the AF content by ∼29,71% in the by-product of ethanol production (DDGS) (Figure 8). DDGS is characterized by its high contents of protein, fiber, and various minerals and vitamins (Chatzifragkou and Charalampopoulos, 2018; Chen et al., 2019), which are valuable as animal nutrition in feed. Increasing supply and demand for DDGS (Wu and Munkvold, 2008) is expected to be driven by increased bioethanol production (Mohanty and Swain, 2019), which will allow DDGS to be used as a renewable source (Kumar and Singh, 2019). There is still no information offering biological tools for the complete elimination of mycotoxins from fermentation media, including raw materials.

The obtained results of the possible biological decontamination are in agreement with other reviewed papers (Mahmood Fashandi et al., 2018; Chiocchetti et al., 2019). Fermentation is influenced by microorganisms occurring naturally in the raw materials or by the addition of starter cultures of microorganisms. Yeasts, such as *S. cerevisiae* and various lactic acid bacteria (LAB), occur naturally and spontaneously as a natural part of fermentation in the food industry. Detoxification of mycotoxins usually occurs in two stages: sorption and enzymatic degradation of mycotoxins (Perczak et al., 2018; Pereyra et al., 2018).

## Conclusion

A portable acoustic penetration spectrometer was applied for high-throughput monitoring of contaminated corn and nuts (hazelnuts and peanuts). Strong correlation coefficients were achieved between acoustic results and corn grain density and AF concentrations in model systems (*R*^2^ = 0.729 and −0.684, *p* < 0.05, respectively). The relationships between the amplitude of the acoustic signal penetrating the samples and AF contamination of hazelnuts and peanuts presented strong relationships (*R*^2^ = 0.816 and 0.803, *p* < 0.05, respectively). Our results show that bioprocesses such as bioethanol production cannot completely eliminate AF contamination of dried distillers’ grains with solubles (AF removal was on average 29.71%). In addition, there is a problem with bioethanol quality (lower ethanol content and more volatile metabolites). Therefore, the development and use of rapid methods, such as the use of broadband capacitive acoustic transducers, are still very attractive solutions for AF prevention. Because of its speed, non-invasive character, and quantification ability, this method is comparable in precision to wet-chemistry methods such as ELISA and is far faster and cheaper per analysis to set up than wet-chemistry methods. It lends itself to the monitoring and high-throughput detection of AFs in corn and nuts and eliminates their contamination of the food chain.

## Data Availability Statement

All datasets generated for this study are included in the article.

## Author Contributions

GJ, EB, and AS conceived and designed the experiments. GB and KT-R performed the experiments. DC and DZ analyzed the data. GJ and EB contributed the reagents, materials, and analysis tools. GJ, DC, and AS wrote the manuscript. All authors read and approved the final version of manuscript.

## Conflict of Interest

The authors declare that the research was conducted in the absence of any commercial or financial relationships that could be construed as a potential conflict of interest.
